# Gene expression profiling of potential peroxisome proliferator-activated receptor (PPAR) target genes in human hepatoblastoma cell lines inducibly expressing different PPAR isoforms

**DOI:** 10.1186/1478-1336-3-3

**Published:** 2005-10-03

**Authors:** Keisuke Tachibana, Yumi Kobayashi, Toshiya Tanaka, Masayuki Tagami, Akira Sugiyama, Tatsuya Katayama, Chihiro Ueda, Daisuke Yamasaki, Kenji Ishimoto, Mikako Sumitomo, Yasutoshi Uchiyama, Takahide Kohro, Juro Sakai, Takao Hamakubo, Tatsuhiko Kodama, Takefumi Doi

**Affiliations:** 1 Graduate School of Pharmaceutical Sciences, Osaka University, Osaka, Japan; 2 Laboratory for System Biology and Medicine, The Research Center for Advanced Science and Technology, the University of Tokyo, Tokyo, Japan; 3 Perseus Proteomics Inc, Tokyo, Japan; 4 Graduate School of Medicine, Osaka University, Osaka, Japan

## Abstract

**Background:**

Peroxisome proliferator-activated receptors (PPARs) are ligand-activated transcription factors and commonly play an important role in the regulation of lipid homeostasis. To identify human PPARs-responsive genes, we established tetracycline-regulated human hepatoblastoma cell lines that can be induced to express each human PPAR and investigated the gene expression profiles of these cells.

**Results:**

The expression of each introduced PPAR gene was investigated using the various concentrations of doxycycline in the culture media. We found that the expression of each PPAR subtype was tightly controlled by the concentration of doxycycline in these established cell lines. DNA microarray analyses using these cell lines were performed with or without adding each subtype ligand and provided much important information on the PPAR target genes involved in lipid metabolism, transport, storage and other activities. Interestingly, it was noted that while ligand-activated PPARδ induced target gene expression, unliganded PPARδ repressed these genes. The real-time RT-PCR was used to verify the altered expression of selected genes by PPARs and we found that these genes were induced to express in the same pattern as detected in the microarray analyses. Furthermore, we analysed the 5'-flanking region of the human *adipose differentiation-related protein *(*adrp*) gene that responded to all subtypes of PPARs. From the detailed analyses by reporter assays, the EMSAs, and ChIP assays, we determined the functional PPRE of the human *adrp *gene.

**Conclusion:**

The results suggest that these cell lines are important tools used to identify the human PPARs-responsive genes.

## Background

The peroxisome proliferator-activated receptors (PPARs) are ligand-activated transcription factors that belong to the nuclear hormone receptor superfamily [1]. Three subtypes, PPARα, PPARβ/δ and PPARγ, have been identified and these subtypes with a high degree of sequence conservation of each subtype across various species, have been characterized. The DNA binding domains of the three subtypes are 80% identical, while their ligand-binding domains exhibit a lower degree (approx. 65%) of identity. Consistent with this relatively high divergence among the subtype-specific ligand binding domains, differential activation of PPARs by endogenous and exogenous compounds may account for the specific biological activity of the three PPAR subtypes [2,3].

PPARα is expressed in the liver, kidney, heart and muscle where it regulates energy homeostasis. PPARα activates fatty acid catabolism, stimulates gluconeogenesis and ketone body synthesis and is involved in the control of lipoprotein assembly [4]. Although PPARα is well characterized, the functional differences of PPARα derived from species are not clear. For example, sustained PPARα activation has carcinogenic consequences in the liver of rodents, but long-term usage of PPARα activators in epidemiological data, has proven that similar effects are unlikely to occur in humans [5,6]. PPARδ is expressed ubiquitously, and is implicated in fatty acid oxidation, in keratinocyte differentiation and wound healing, and in mediating very low density lipoprotein signalling of the macrophage [7-11]. However, the function of PPARδ is less understood than PPARα and γ. There are two PPARγ isoforms, PPARγ1 and γ2. PPARγ2, which is generated by alternative splicing, contains an additional 28 amino acids at the N-terminal end relative to PPARγ1. PPARγ3 is a splicing variant of PPARγ1 and gives rise to the same protein. PPARγ2 is expressed exclusively in adipose tissue and has a pivotal role in adipocyte differentiation, lipid storage in the white adipose tissue and energy dissipation in the brown adipose tissue [12,13]. On the other hand, PPARγ1 is expressed in the liver and other tissues, and the expression of hepatic PPARγ is increased in some obese and diabetic model mice [14-17]. PPARγ is involved in glucose metabolism through the improvement of insulin sensitivity; however, its function is not well defined.

All PPARs bind to a direct repeat of two hexanucleotides, spaced by one or two nucleotides (the DR1 or DR2 motif) as heterodimers with the retinoid X receptor (RXR), and activate several target genes [18-20]. These peroxisome proliferator responsive elements (PPREs) are found in various genes that are involved in lipid metabolism and energy homeostasis, including lipid storage or catabolism, and fatty acid transport, uptake and intracellular binding [21].

One of the approaches for investigating target genes of PPARs is to construct stable cell lines that can be induced to express PPARs. The tet-off system is a well-established system for inducible gene expression. In this system, transcription is turned on or off in response to doxycycline (Dox; a tetracycline derivative) in a strictly dose-dependent manner. Therefore, background or leaky expression in the absence of induction is extremely low. The tet-off system enables us to compare the same cell line before and after induction of the gene of interest. Previously, we established the human hepatoblastoma cell lines (HepG2 cells) which were strictly induced to express the hepatitis C non-structural proteins by removing Dox from the media, and we characterized the changes in mRNA expression profile using DNA microarray analyses [22].

In the present study, to identify human PPARs-responsive genes in the liver cell line, we established tightly tet-regulatable HepG2 cells which can be induced to express each human PPAR. We demonstrated that human PPARs are important regulators of lipid homeostasis in these cell lines using DNA microarray and real-time RT-PCR technologies. Subsequent analyses revealed that all PPARs induced human *adipose differentiation-related protein *(*adrp*) gene expression through the same PPRE of the ADRP promoter, while unliganded PPARδ repressed this gene. Our results imply that these cell lines are important tools, which can be used to identify the human PPARs-responsive genes.

## Results

### Establishment of HepG2 cells that can be induced to express PPARs

**Figure 1:**
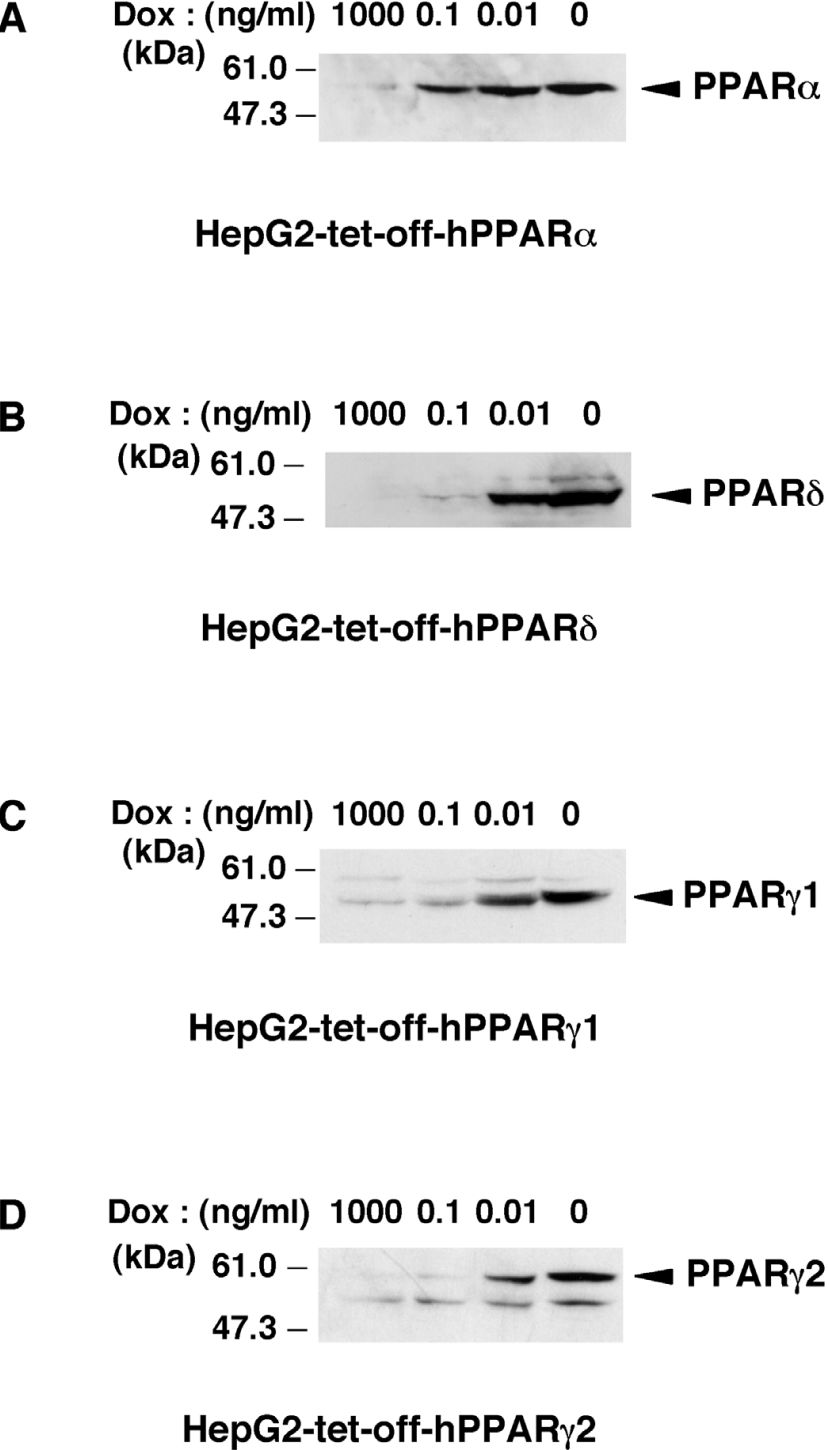
**Induction of the expression of PPAR by doxycycline in established cell lines. ***A*, *B*, *C *and *D*, Nuclear extracts (50 μg protein/lane) from each cell line cultured in the presence of the indicated amounts of Dox for 5 days were subjected to SDS-PAGE and immunoblots were performed with anti-PPARα (A), anti-PPARδ (B) or anti-PPARγ (C and D).

**Figure 2:**
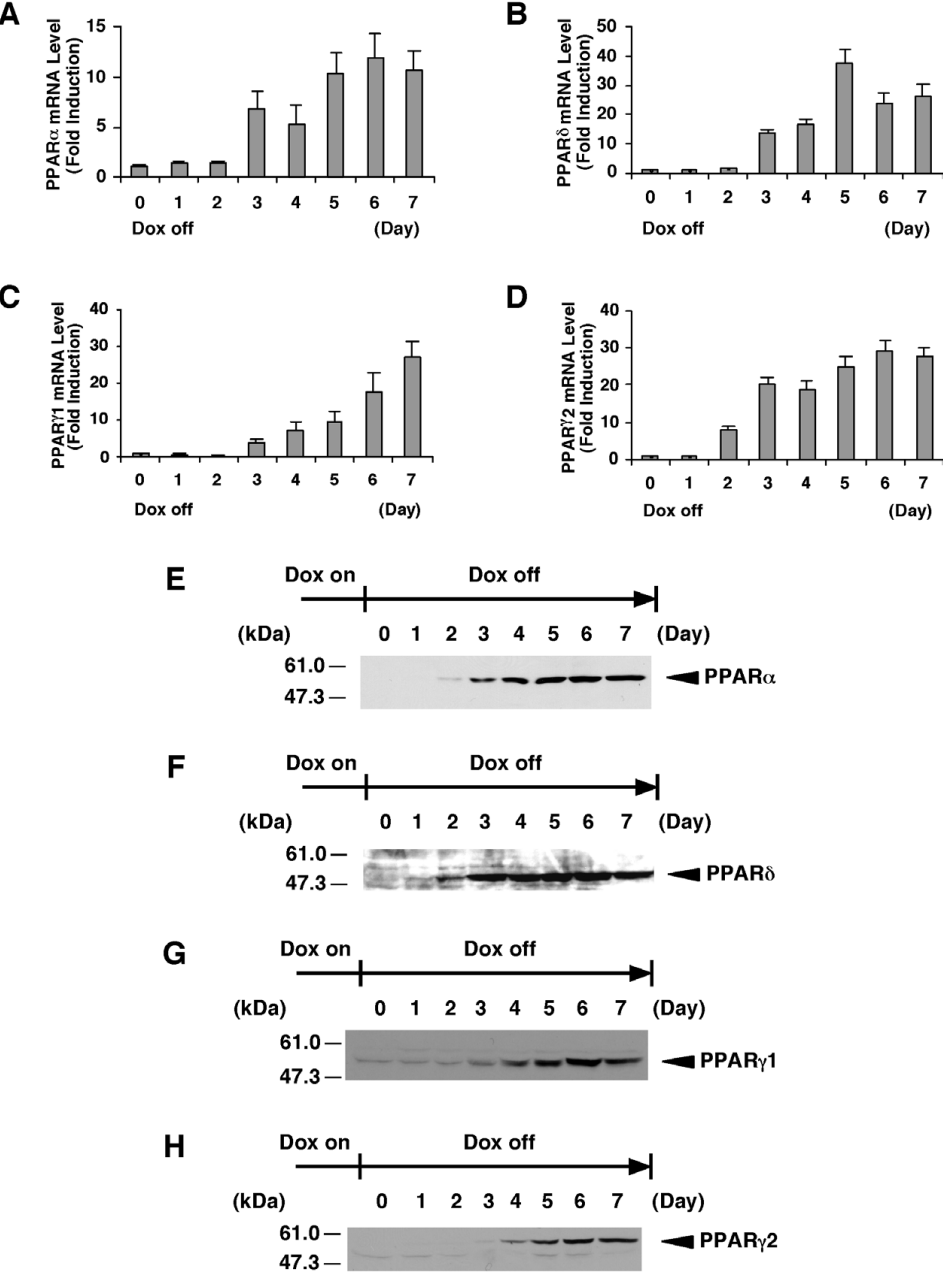
**Time course of PPAR expression in established cell lines. ***A*, *B*, *C *and *D*, The cell lines for the expression of PPARα (A), PPARδ (B), PPARγ1 (C) or PPARγ2 (D) were cultured in the presence of Dox. At time point 0, Dox was removed from the medium. The total RNAs were extracted from the cells cultured for the indicated days after removal of Dox and the amount of mRNAs of PPARs were measured by real time RT-PCR. Values are expressed as the mean ± S.E.M. (n = 3), relative to the control (0 day) set as 1. *E*, *F*, *G *and *H*, Nuclear extracts (50 μg protein/lane) from the cell lines expressing PPARα (E), PPARδ (F), PPARγ1 (G) or PPARγ2 (H) cultured for the indicated days after removal of Dox were subjected to SDS-PAGE and immunoblots were performed with anti-PPARα (E), anti-PPARδ (F) or anti-PPARγ (G and H).

To identify human PPARs-responsive genes, we established each cell line that expresses any one of the PPAR subtypes (α, β/δ, γ1 or γ2) using the tet-regulated system. Previously, we established the tightly tet-regulatable HepG2 cell clone (HY-Toff) which was transfected with the pTet-off vector, and that this clone had a large induction/repression rate [22]. The tet-off regulatable HY-Toff cells were transfected with the pBabepuro (for puromycin resistance) and pBI-EGFP vector harbouring the cDNA for human PPARα, PPARδ, PPARγ1 or PPARγ2. We picked out puromycin-resistant clones. Among these clones, we selected the strictly responsive cell lines (HepG2-tet-off-hPPAR cells) to the concentration of Dox. These cell lines were cultured in the presence of Dox at 0, 0.01, 0.1 or 1000 ng/ml for 5 days, and we found that PPARs expression in these cell lines was induced in a dose-dependent manner (Figure 1A–D). We then examined the time course experiment of each PPAR expression after the removal of Dox. The results showed that both the mRNA (Figure 2A–D) and protein (Figure 2E–H) levels of each PPAR were increased after the removal of Dox from the culture medium. Under these conditions, cell proliferation in these cell lines was not affected until 7 days after the removal of Dox. Based on these results, we decided that the expression of each PPAR on the 5th day was suitable for further analyses, such as the determination of the target genes of each PPAR subtype.

### Changes in mRNA expression profiles by the induction of each PPAR subtype with the PPAR ligands

To characterize the regulation of gene expression by each PPAR subtype, we performed oligonucleotide microarray analyses. HepG2-tet-off-hPPAR cells were cultured in the presence or absence of Dox for 5 days, then treated with the PPAR ligands (100 μM fenofibric acid for PPARα, 100 nM GW501516 for PPARδ or 10 μM ciglitizone for PPARγ) or vehicle for 24 h. Total RNA samples were prepared from these cells, and oligonucleotide microarray analyses were performed using the Affymetrix HG-U133A arrays that contain >22,000 probe sets.

To identify human PPARs-responsive genes, we analysed the genes induced by ligand-activated PPARs. The criteria for selecting genes are described in the Methods section. Based on these criteria, 29, 21, 60 and 107 genes were up-regulated by ligand-activated PPARα, PPARδ, PPARγ1 and PPARγ2, respectively. As shown in Table 1 and the additional file 1, each PPAR affects the expression levels of several genes involved in lipid metabolism, glucose homeostasis, etc. PPARα tends to induce the expression of a number of genes involved in the β-oxidation of fatty acids by mitochondria, peroxisomal fatty acid oxidation, antioxidant and ketogenesis. Most of the genes that were induced by PPARδ were similar to those exhibited by PPARα. We observed that induced expression of PPARδ increased the expression in the presence of the ligand, while unliganded PPARδ repressed the expression of its target genes instead. On the other hand, PPARγ tends to induce the expression of several genes involved in gluconeogenesis, lipid storage, transport and metabolism. Moreover, PPARγ is likely to induce several genes involved in angiogenesis, cytoskeleton organization, protein modification, regulation of transcription, signal transduction, etc.

**Table 1:** Changes in mRNA expression levels of metabolism-related genes in HepG2-tet-off-hPPAR cells by ligands. Microarray analyses were performed on HepG2-tet-off-hPPAR cells; the cells were cultured in the presence (Dox) or absence of Dox for 5 days. In the absence of Dox, the cells were treated with PPAR ligands (100 μM fenofibric acid for PPARα (Feno), 100 nM GW501516 for PPARδ (GW) or 10 μM ciglitizone for PPARγ (Cig)) or vehicle (DMSO) for 24 h. Gene expression profiles were compared between DMSO and Dox (DMSO *versus *Dox), ligands and Dox (Feno *versus *Dox, GW *versus *Dox, and Cig *versus *Dox were indicated in the case of PPARα, PPARδ and PPARγ, respectively) or ligands and DMSO (Feno *versus *DMSO, GW *versus *DMSO, and Cig *versus *DMSO were indicated in the case of PPARα, PPARδ and PPARγ, respectively). Samples were analysed using GeneChip^® ^software Microarray Suite (MAS) Ver.5.0 (Affymetrix).

Gene	Genbank accession No.	PPARα			PPARδ			PPARγ1			PPARγ2		
		
		Fold change			Fold change			Fold change			Fold change		
		
		DMSO vs. Dox	Feno vs. Dox	Feno vs. DMSO	DMSO vs. Dox	GW vs. Dox	GW vs. DMSO	DMSO vs. Dox	Cig vs. Dox	Cig vs. DMSO	DMSO vs. Dox	Cig vs. Dox	Cig vs. DMSO
Fatty acid metabolism													
acyl-CoA synthetase long-chain family member 1	NM_001995	1.84	3.49	1.90	0.82	1.72	2.09	0.88	2.30	2.60	0.82	1.00	1.23
carnitine palmitoyltransferase 1A (liver)	NM_001876	1.48	2.86	1.93	0.37	2.05	5.60	0.74	1.35	1.83	1.12	1.24	1.10
solute carrier family 25 (carnitine/acylcarnitine translocase), member 20	NM_000387	1.20	2.23	1.85	0.91	1.36	1.50	0.83	0.93	1.12	1.04	1.27	1.22
acyl-Coenzyme A dehydrogenase, very long chain	NM_000018	2.16	3.46	1.60	0.92	2.69	2.93	1.36	1.97	1.45	1.55	2.33	1.50
acyl-Coenzyme A dehydrogenase, C-4 to C-12 straight chain	NM_000016	1.62	2.52	1.56	0.64	2.03	3.16	1.24	1.48	1.20	1.40	1.80	1.29
hydroxyacyl-Coenzyme A dehydrogenase/3-ketoacyl-Coenzyme A thiolase/enoyl-Coenzyme A hydratase (trifunctional protein), alpha subunit	NM_000182	1.48	2.43	1.65	0.89	1.49	1.66	1.34	1.62	1.21	1.41	2.29	1.62
hydroxyacyl-Coenzyme A dehydrogenase/3-ketoacyl-Coenzyme A thiolase/enoyl-Coenzyme A hydratase (trifunctional protein), beta subunit	NM_000183	1.74	2.66	1.53	0.95	1.30	1.36	1.17	1.49	1.27	1.14	1.62	1.42
acetyl-Coenzyme A acyltransferase 2 (mitochondrial 3-oxoacyl-Coenzyme A thiolase)	NM_006111	1.58	1.92	1.21	1.06	1.63	1.53	0.89	1.31	1.47	1.18	2.44	2.06
acetyl-Coenzyme A acyltransferase 1 (peroxisomal 3-oxoacyl-Coenzyme A thiolase)	NM_001607	1.42	1.62	1.14	0.68	1.47	2.15	1.22	2.07	1.70	1.42	2.01	1.41
enoyl Coenzyme A hydratase 1, peroxisomal	NM_001398	2.28	2.61	1.15	0.99	2.05	2.07	1.16	1.46	1.26	1.44	1.98	1.38
Antioxidant													
catalase	NM_001752	1.51	2.36	1.56	0.68	1.45	2.13	1.06	1.54	1.45	1.69	2.59	1.53
vanin 1	NM_004666	5.07	12.92	2.55	0.68	1.91	2.80	2.42	6.02	2.49	1.77	5.70	3.23
Ketogenesis													
3-hydroxy-3-methylglutaryl-Coenzyme A synthase 2 (mitochondrial)	NM_005518	32.40	156.62	4.83	1.13	2.51	2.22	2.14	6.57	3.07	2.20	6.53	2.96
Transport/strage													
fatty acid binding protein 1, liver	NM_001443	1.39	2.08	1.50	0.18	1.33	7.57	2.73	6.52	2.39	3.52	10.44	2.96
adipose differentiation-related protein	NM_001122	1.96	3.44	1.75	0.22	2.90	13.02	3.33	6.06	1.82	3.40	5.73	1.68
C-terminal linking and modulating protein/PDZ domain containing 1	NM_002614	2.16	3.02	1.40	0.52	2.85	5.45	1.03	2.42	2.34	1.98	3.21	1.62
lipase, hepatic	NM_000236	8.34	10.39	1.25	0.72	2.89	4.00	1.08	2.47	2.28	1.64	3.99	2.44
Gluconeogenesis													
aquaporin 3	NM_004925	3.00	5.86	1.95	0.26	1.91	7.38	1.58	3.41	2.16	1.38	3.09	2.24
glycerol kinase	NM_000167	0.87	1.57	1.79	0.73	0.79	1.08	1.00	1.21	1.21	1.84	3.17	1.73
phosphoenolpyruvate carboxykinase 1 (soluble)	NM_002591	6.39	17.90	2.80	0.83	4.64	5.56	6.94	29.19	4.20	34.46	110.78	3.21
Metabolism													
angiopoietin-like protein 4	NM_016109	1.71	17.97	10.54	3.00	25.67	8.56	1.24	7.27	5.84	3.88	33.56	8.66
heme oxygenase (decycling) 1	NM_002133	1.25	2.21	1.77	0.66	1.25	1.90	1.31	2.20	1.68	2.18	4.20	1.93
biliverdin reductase B (flavin reductase (NADPH))	NM_000713	1.07	1.65	1.55	0.84	0.86	1.03	0.87	1.58	1.81	1.36	4.03	2.97
sulfotransferase family, cytosolic, 2A, dehydroepiandrosterone (DHEA) -preferring, member 1	NM_003167	2.61	2.86	1.10	0.70	2.08	2.98	0.95	1.76	1.85	1.71	1.68	0.98
abhydrolase domain containing 3	NM_138340	1.68	3.51	2.08	0.54	2.16	3.96	1.76	3.30	1.88	2.01	3.99	1.99

### Verification of gene expression profiles with quantitative PCR analyses

**Figure 3:**
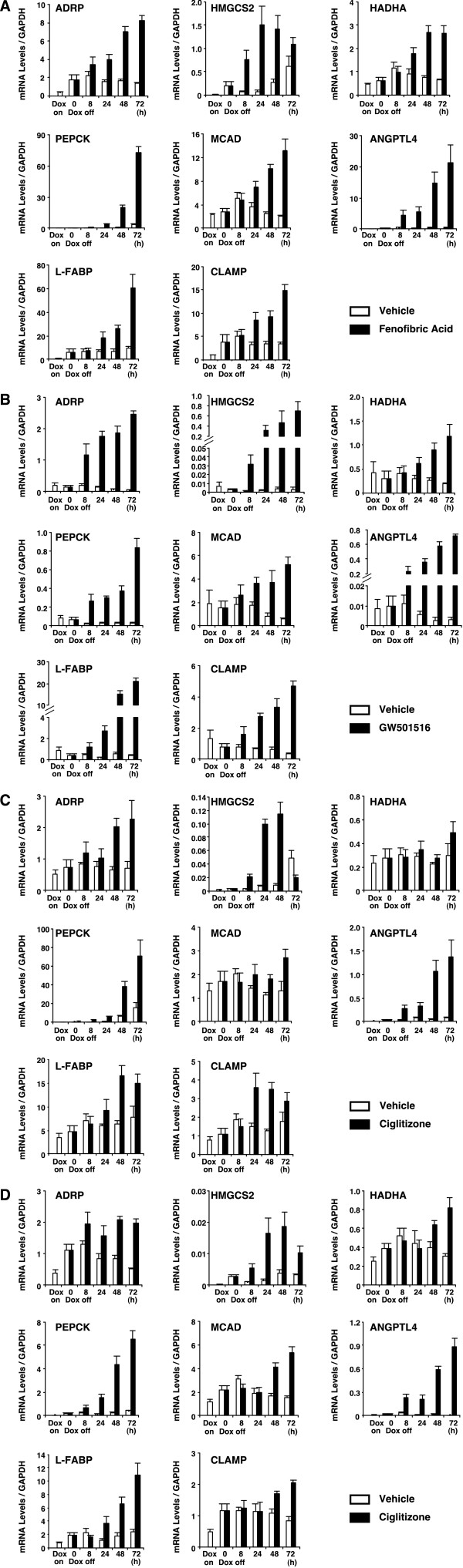
**Eight gene expressions were modulated by PPARs in tet-regulated HepG2 cells. ***A*, *B*, *C *and *D*, HepG2-tet-off-hPPAR cells were treated with DMSO (vehicle) or PPAR ligands (100 μM fenofibric acid for PPARα (A), 100 nM GW501516 for PPARδ (B), 10 μM ciglitizone for PPARγ1 (C) or PPARγ2 (D)) for 0, 8, 24, 48 or 72 h in the absence of Dox. Messenger RNA levels of human ADRP, HMGCS2, HADHA, PEPCK, MCAD, ANGPTL4, L-FABP and CLAMP/PDZK1 were measured by real time RT-PCR. Values are expressed as mean ± S.E.M. (n = 3) target mRNA levels normalized to GAPDH mRNA.

The expression of a subset of genes in response to treatment with PPAR ligands was further characterized using real-time quantitative PCR analyses. Total RNA samples were prepared from HepG2-tet-off-hPPAR cells, which were cultured in the presence or absence of Dox for 5 days, and subsequently treated with the PPAR ligands (100 μM fenofibric acid, 100 nM GW501516 or 10 μM ciglitizone for PPARα, PPARδ and PPARγ, respectively) or vehicle for 0, 8, 24, 48 or 72 h. Several PPAR-responsive genes (ADRP, 3-hydroxy-3-methylglutaryl-Coenzyme A synthase 2 (mitochondrial) (HMGCS2), hydroxyacyl-Coenzyme A dehydrogenase/3-ketoacyl-Coenzyme A thiolase/enoyl-Coenzyme A hydratase (trifunctional protein), α subunit (HADHA), phosphoenolpyruvate carboxykinase 1 (PEPCK), acyl-Coenzyme A dehydrogenase, C-4 to C-12 straight chain (MCAD), angiopoietin-like protein 4 (ANGPTL4), fatty acid binding protein 1, liver (L-FABP)) were up-regulated by the expression of PPARα and PPARγ (Figure 3). On the other hand, only ligand-activated PPARδ induced the expression of these genes, but unliganded PPARδ repressed them, in agreement with microarray analyses (Figure 3B and Table 1).

Although until now C-terminal linking and modulating protein/PDZ domain containing 1 (CLAMP/PDZK1) mRNA has not been reported as a target for PPARs, we observed that PPARs induced CLAMP/PDZK1 mRNA by microarray analyses (Table 1). We, therefore, verified the expression of CLAMP/PDZK1 mRNA using real-time quantitative PCR analysis. A similar result was observed on the expression pattern of CLAMP/PDZK1 mRNA (Figure 3).

Although all subtypes of PPARs could induce the expression of these genes, PPARα was more effective than other subtypes in the real-time PCR analyses (Figure 3).

### PPARs modulate human ADRP promoter activity via a PPRE

**Figure 4:**
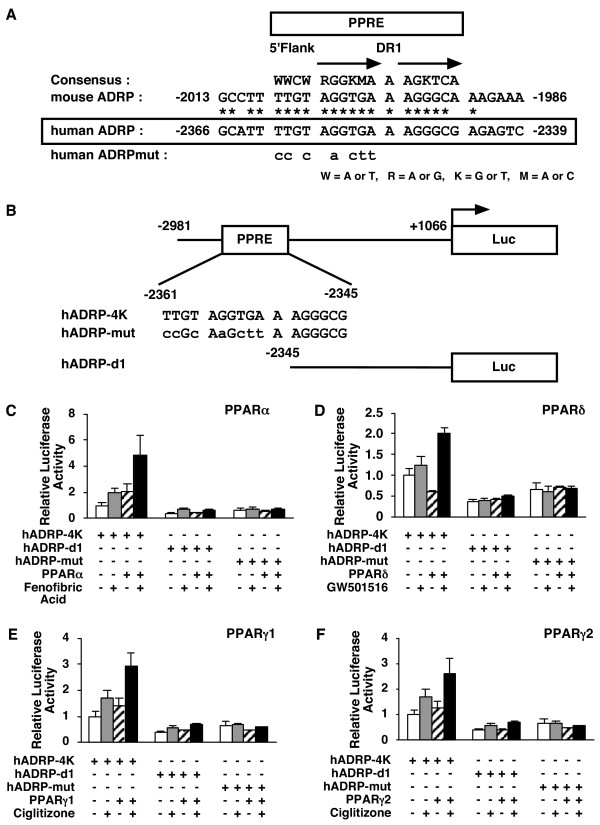
**PPARs modulate human ADRP promoter activity via a PPRE located between -2361 and -2345 bp. ***A*, A sequence corresponding to the -2366/-2339 region of the human *adrp *gene, was compared with a consensus PPRE and the analogous regions in the mouse *adrp *gene promoter (-2013/-1986). A *human ADRPmut *indicates a human ADRP promoter whose potential PPRE was mutated. *Asterisks *denote conserved bases and *arrows *represent response element half-sites. *B*, Schematic representation of the chimeric genes containing the human ADRP promoters; each wild type (hADRP-4K), point mutation (hADRP-mut), and deletion (hADRP-d1) of the ADRP promoter was cloned in front of the firefly luciferase reporter gene. *Lowercase letters *indicate mutations introduced in the human ADRP PPRE. *C*, *D*, *E *and *F*, HepG2 cells were co-transfected with a human ADRP reporter plasmid (50 ng), phRL-TK (50 ng) and either pcDNA3-hPPARα (5 ng) (C), pcDNA3-hPPARδ (5 ng) (D), pcDNA3-hPPARγ1 (5 ng) (E) or pcDNA3-hPPARγ2 (5 ng) (F). Transfected cells were treated with ligands (100 μM fenofibric acid (C), 100 nM GW501516 (D) or 10 μM ciglitizone (E and F)) for 24 h and the cells were used for reporter gene assays. Luciferase activities from reporter plasmids were normalized by internal *Renilla *luciferase activity. Values are expressed as fold induction of the control (the value when only reporter plasmid (ADRP-4K) was transfected) set at 1. Values represent the mean ± S.E.M. (n = 3).

To determine whether one of these genes was regulated by PPARs directly, we performed transient transfection experiments with the human ADRP promoter. It has been reported that the mouse ADRP promoter contains a functional PPRE [11]. Examination of the corresponding region of the human *adrp *gene indicated that the essential features of this element are likely to be conserved, and we found that the human ADRP promoter contained a potential PPRE at positions -2361 to -2345 (Figure 4A).

To analyse whether this site can be regulated by PPARs, either the wild type 4-kb fragment of the human ADRP promoter (hADRP-4K), deletion promoter (hADRP-d1) whose potential PPRE is deleted or mutation promoter (hADRP-mut) whose potential PPRE is mutated, was cloned in front of the pGL3-luciferase reporter gene to construct reporter plasmids (Figure 4B). We co-transfected either the PPAR expression plasmid (pcDNA3-hPPARα, pcDNA3-hPPARδ, pcDNA3-hPPARγ1 or pcDNA3-hPPARγ2) or the vector plasmid (pcDNA3) as a control together with each reporter plasmid into HepG2 cells, and subsequently incubated these cells with or without ligands (100 μM fenofibric acid, 100 nM GW501516 or 10 μM ciglitizone for PPARα, PPARδ and PPARγ, respectively) for 24 h. Ligand-activated PPARs induced the promoter activity of the wild type 2 to 5-fold higher than the control (no PPAR expression without ligand) (Figure 4C–F). Interestingly, the expression of PPARδ without ligand diminished the wild type ADRP reporter gene expression to about 60% of the control in HepG2 cells (Figure 4D). We did not detect this observation when either deleted or mutated PPRE reporter plasmid was used. These data indicate that this PPRE in the ADRP promoter is a *cis*-acting element by which PPARs modulate human ADRP promoter activity.

**Figure 5:**
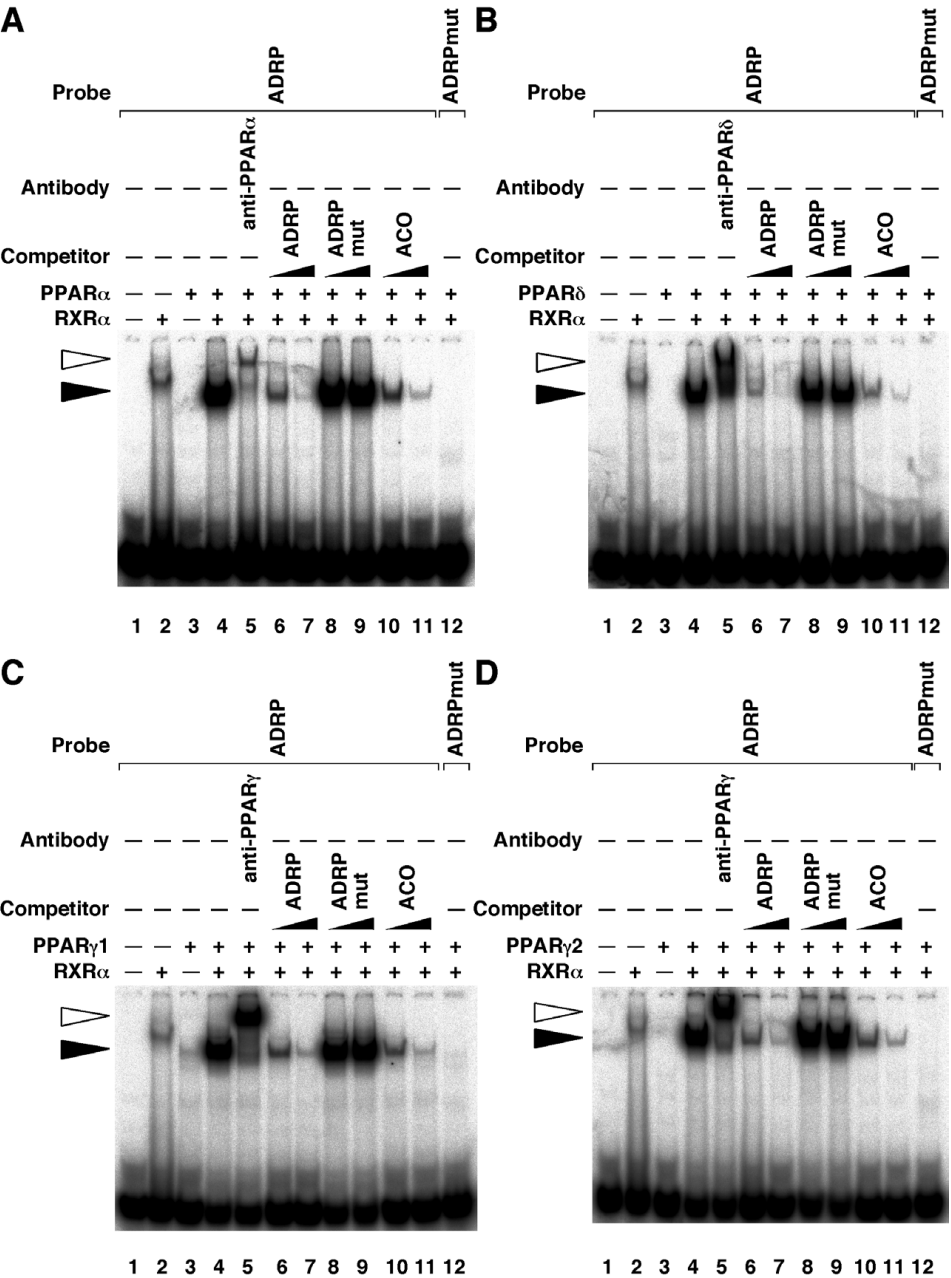
**PPARs bind to the PPRE in the -2366/-2339 region of the human *adrp *gene. ***A*, *B*, *C *and *D*, EMSAs were performed with ^32^P-labelled either ADRP or mutated ADRP (ADRPmut) oligonucleotides in the presence of purified PPARα (A), purified PPARδ (B), *in vitro *transcribed/translated PPARγ1 (C), purified PPARγ2 (D) and/or purified RXRα proteins. Supershift experiments were carried out using anti-PPARα (A), anti-PPARδ (B) or anti-PPARγ (C and D) antibodies. Unlabelled oligonucleotides (ADRP, ADRPmut or ACO) were used at 10- or 100-fold molar excess to the labelled probe to perform competition assays. *Closed *and *open arrowheads *indicate the specific bands and the supershift bands, respectively.

To determine whether human PPARs/RXRα heterodimers bind this PPRE in the ADRP promoter, EMSAs were performed using this response element as a radiolabelled probe (Figure 5A–D). EMSAs revealed that all PPAR subtypes could bind the PPRE of the ADRP promoter in the presence of RXRα (closed arrowhead on lanes 2 to 4). Furthermore, these complexes were supershifted by anti-PPAR antibodies (open arrowhead on lane 5). This complex formation was competed by increasing amounts (10- and 100-fold excess) of unlabeled self-competitor (ADRP) and rat acyl CoA oxidase (ACO) PPRE fragments, but not by the mutated PPRE (ADRPmut) probes (lanes 6 to 11). Furthermore, no protein-DNA complex was observed when using the mutated PPRE (ADRPmut) probe (lane 12). Taken together, these data demonstrate that all PPARs bind to the same PPRE site at the position -2361 to -2345, and modulate human ADRP promoter activity.

**Figure 6:**
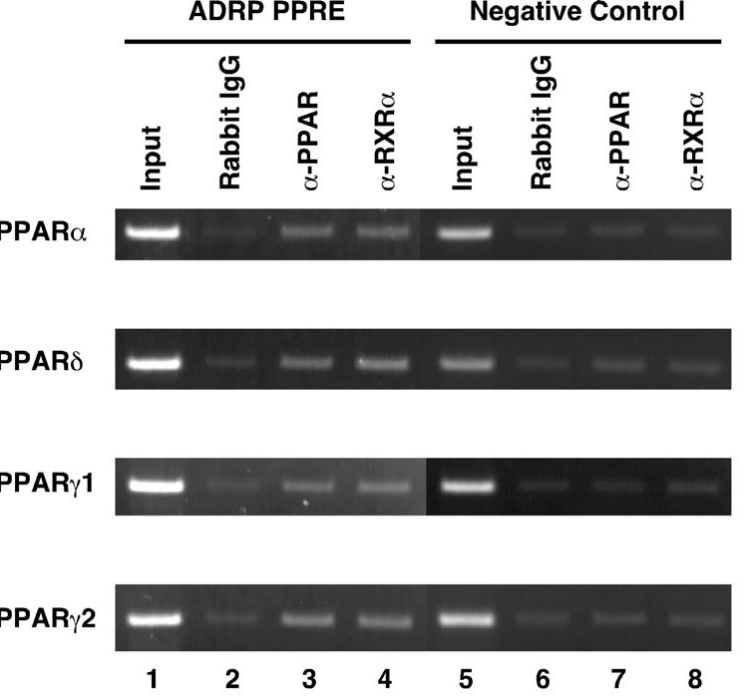
**Chromatin immunoprecipitation assays of the ADRP promoter. **HepG2-tet-off-hPPAR cells were treated with PPAR ligands (100 μM fenofibric acid for PPARα, 100 nM GW501516 for PPARδ, or 10 μM troglitazone for PPARγ) for 8 h in the absence of Dox and processed for the ChIP assays. Soluble chromatin was immunoprecipitated with pre-immune rabbit IgG (lanes 2 and 6), anti-PPAR antibodies (lanes 3 and 7), or anti-RXRα antibody (lanes 4 and 8). Immunoprecipitates were subjected to PCR with a primer-pair specific to the ADRP promoter. As a negative control, a second set of primers were used to amplify another genomic region that was not expected to interact with the PPARs. PCR was performed with total chromatin input (lanes 1 and 5).

To confirm that the PPARs/RXRα heterodimers bind to the PPRE in the ADRP promoter *in vivo*, we performed chromatin immunoprecipitation (ChIP) assays using an anti-PPARα, anti-PPARδ, anti-PPARγ, or anti-RXRα antibody. HepG2-tet-off-hPPAR cells were cultured in the absence of Dox for 5 days, and were subsequently treated with the PPAR ligands (100 μM fenofibric acid, 100 nM GW501516 or 10 μM troglitazone for PPARα, PPARδ and PPARγ, respectively) for 8 h and subsequently used for ChIP assays. PPARs/RXRα heterodimers bound to the PPRE in the ADRP promoter *in vivo *(Figure 6).

## Discussion

In the present study, in order to identify human PPARs-responsive genes in the liver, we have established tightly tet-regulatable human hepatoblastoma cell lines that can be induced to express each human PPAR (HepG2-tet-off-hPPAR cells). DNA microarray and real-time RT-PCR analyses using these stable cell lines indicate that PPARs activate gene expression involved in lipid metabolism, glucose homeostasis, and other activities (Table 1 and Figure 3). Cell proliferation of these cell lines was not affected until 7 days after removal of Dox. However, further investigation is required to define the influence of the expression of PPARs on the proliferation of these cell lines.

We identified a series of genes that are critical for many aspects of carbohydrate and lipid metabolism, including intracellular fatty acid transport, mitochondrial fatty acid β-oxidation, and ketogenesis by PPARα (Table 1). Indeed, PPARα regulates the expression of genes that encode for enzymes involved in peroxisomal proliferation and fatty acid oxidation in peroxisomes and mitochondria [2]. In addition, PPREs are present in the promoter region or the intronic sequence of these target genes [4]. However, our results show that PPARα and other PPAR subtypes induce expression of these genes. In fact, liver PPARγ regulated the expression levels of genes involved in lipogenesis, fatty acid transport, storage, and oxidation using both wild type mice and adipose-deficient mice [23]. Other reports also showed that PPARδ induced fatty acid oxidation by regulating genes involved in fatty acid transport, β-oxidation, and mitochondrial respiration in various tissues [7-9]. These reports support our results whereby each PPAR subtype induces fatty acid oxidation in the hepatoblastoma cell lines. In the real-time PCR analyses PPARα was more effective than other subtypes in the hepatoblastoma cell lines, although all subtypes could induce the expression of these genes (Figure 3). Thus, we assume that PPARα plays a major role in the liver, however, the mechanism of this effect remains uncertain.

Mammalian cells use glucose as a major energy source, and the origin of hepatic glucose production shifts from mainly glycogenolysis to gluconeogenesis as fasting prolongs to maintain blood glucose levels. The main precursors for hepatic gluconeogenesis are lactate, pyruvate, glycerol and alanine, which are converted into glucose via a series of reactions in the cytosol and mitochondria. PPARα plays a pivotal role in the management of energy stores during fasting [2]. Indeed, PPARα-null mice, fasted for 24 h, exhibit severe hypoglycaemia [24,25]. Recently, Patsouris *et al. *demonstrated that PPARα stimulates the expression of a set of genes involved in hepatic gluconeogenesis from glycerol [26]. In the present study, glycerol kinase and glycerol transporter aquaporin 3 were up-regulated by the liganded PPARs. Surprisingly, an unexpected finding was that the PEPCK was up-regulated by PPARs in these cell lines. PEPCK catalyses the rate-limiting step in liver gluconeogenesis. Way *et al. *showed that when Zucker diabetic fatty rats were treated with PPARγ agonists, PEPCK expression was decreased in the liver, and suggested that PPARγ agonists decrease gluconeogenesis [27]. Although we cannot fully explain this inconsistency, there are several reports, which support our results. An *in vivo *study showed that PEPCK mRNA levels are elevated in the livers of dexamethasone (DEX)-treated PPARα+/+ mice, and not DEX-treated PPARα-/- mice, and that PEPCK is increased when hepatic PPARα expression is reconstituted in DEX-treated PPARα-/-LDLR-/- mice. Furthermore, the combination of DEX and the potent PPARα ligand Wy14,643 increased the expression of PEPCK in human hepatocytes [28]. However, the reason for this differential induction remains unknown.

CLAMP, a four-PDZ-domain-containing protein also called PDZK1 [29], was identified as the scavenger receptor class B type I (SR-BI)-associated protein from rat liver membrane extracts [30]. CLAMP/PDZK1 regulates the stable SR-BI protein expression by a post-transcriptional mechanism [31]. The hepatic expression of SR-BI plays a critical role in lipoprotein metabolism, mainly due to its ability to mediate selective high density lipoproteins (HDL) cholesterol uptake [32,33]. HDL removes cholesterol from peripheral tissues and then transfers it to the liver. CLAMP/PDZK1 may modulate the intracellular transport and metabolism of cholesteryl esters taken up from HDL. Although until now, *clamp/pdzk1 *has not been reported as a direct PPAR target gene, we observed that CLAMP/PDZK1 mRNA was induced by PPARs in the established cell lines (Table 1 and Figure 3). These data suggest that CLAMP/PDZK1 is a target gene of PPARs. In the case of humans, PPRE exists in the CLAMP/PDZK1 promoter and PPARs can directly bind to this region (unpublished data). Therefore, PPAR activators may play a role in the regulation of HDL metabolism. However, further investigations are required to define the molecular mechanisms underlying the PPAR-mediated HDL metabolism.

Our microarray analyses indicated that there are many genes which are induced by all PPAR subtypes (Table 1, additional file 1). However, PPARα and PPARδ tend to induce the expression of a number of genes involved in the β-oxidation of fatty acids by mitochondria and peroxisomal fatty acid oxidation, and PPARγ tends to induce the expression of several genes involved in gluconeogenesis, lipid storage, transport and metabolism. Interestingly, we also observed that the genes involved in angiogenesis, cytoskeleton organization, signal transduction, protein modification and regulation of transcription were up-regulated in our HepG2-tet-off-hPPARγ cell lines. Indeed, there are several reports that PPARγ ligands modulate angiogenesis [34]. Further investigation is necessary to certify the subtype specific function of PPARs.

In the present study, we also observed that ligand-activated PPARδ induced target gene expression, however, unliganded PPARδ repressed these genes in the established cell lines (Table 1, Figures 3, 4). It has been reported that unliganded PPARδ binds to PPRE and recruits corepressors, such as a silencing mediator for retinoid and thyroid hormone receptor (SMRT), nuclear receptor corepressor (NCoR) etc. On the other hand, liganded PPARδ is thought to release the corepressor and form a complex with coactivators [35,36]. From this viewpoint, these established cell lines have a potential application to provide a high-throughput screening to detect the PPARδ ligands (see below).

To further investigate the regulation of target gene expression by PPARs, we analysed the human ADRP promoter in detail. ADRP, expressed ubiquitously, is a protein covering lipid droplets [37]. The mouse *adrp *gene exhibits a PPRE between -2004 and -1992 bp that binds PPARs/RXR heterodimers [11]. The sequence of the corresponding response element in the human gene between -2361 and -2345 bp is very similar to the mouse PPRE (Figure 4A). We performed EMSAs with this element and all PPAR subtypes bound to the same PPRE site of the ADRP promoter (Figure 5). Moreover, this human PPRE site confers transactivation by PPARs to a luciferase reporter gene, and mutations that disrupt the binding sequence of PPARs/RXR on this PPRE abolish transactivation (Figure 4). During the preparation of this manuscript, it was reported that the ADRP PPRE was the functional PPAR binding site and could be activated by human PPARα and δ [38]. In the present study, we showed the same result and that PPARγ1 and γ2 can also bind to PPRE and regulate the expression of the human *adrp *gene. Moreover, we were the first to confirm that PPARs/RXRα heterodimers bind to the PPRE in the ADRP promoter *in vivo *using ChIP assays (Figure 6).

PPARs are linked to metabolic disorders and, therefore, are interesting pharmaceutical targets. Among the synthetic ligands that activate these receptors, the fibrates are hypolipidemic compounds that activate PPARα. The thiazolidinediones, which selectively activate PPARγ, are hypoglycaemic molecules that are used to treat type II diabetes. Recently, we and other groups reported that PPARδ agonists might form effective drugs for obesity, diabetes, and cardiovascular disease [7-9,39,40]. From this viewpoint, these established cell lines have a potential application in various high-throughput assays. For instance, the reporter gene assay using the reporter plasmid containing the ADRP promoter would provide a useful detection system of PPARδ ligands as potential drug candidates in the HepG2-tet-off-hPPARδ cell line [41].

## Conclusion

In conclusion, we established tightly tet-regulatable human hepatoblastoma cell lines that can be induced to express each human PPAR. These cell lines provided evidence that human PPARs are important regulators of lipid and glycerol homeostasis and new insights about the candidate target genes of PPARs. Therefore, these cell lines are powerful tools for analysing the function of human PPARs.

## Methods

### Materials

Fenofibric acid and GW501516 were synthesized as described previously [7]. Ciglitizone was purchased from Sigma. Troglitazone was purchased from Cayman Chemical.

### Plasmid Constructs

Construction of pcDNA3-hPPARα, pcDNA3-hPPARδ, pcDNA3-hPPARγ1 and pcDNA3-hPPARγ2 expression plasmids were described previously [42]. Human PPARs fragments were excised from pcDNA3-hPPAR vectors. pBI-EGFP (Clontech) was digested with *Pvu*II, and ligated with the PPAR fragment (termed pBI-EGFP-hPPAR).

To generate human ADRP promoter-reporter plasmids, human ADRP promoter containing -2981 to +1066 bp (hADRP-4K) and the deletion promoter containing -2345 to +1066 bp (hADRP-d1) were obtained by means of PCR with the bacterial artificial chromosome (BAC) clone plasmid (BACPAC resource center at Children's Hospital Oakland Research Institute) using a forward primer 5'-GGTACCTATCCCTGGTGCCAAAAAGGTTGGGGA-3' (including a *Kpn*I site, underlined) for hADRP-4K or a forward primer 5'-GGTACCGAGAGTCTTCTGATGCAAAGTAAGAGG-3' (including a *Kpn*I site, underlined) for hADRP-d1 and a reverse primer 5'-AGATCTTTTTCTTCCTGGAGAAAGAAATCTGCAGAAAAGAG-3' (including a *Bgl*II site, underlined). Each promoter was cloned into the *Kpn*I-*Bgl*II sites of a pGL3-Basic vector (Promega). A point mutation was introduced into the ADRP promoter by PCR methodology. To generate a point-mutation construct (pGL3-hADRP-mut), a 101 bp mutant fragment was generated from forward primer (5'-GCAAAAAGAAGCTTGCTCAG-3') and reverse primer (5'-GACTCTCGCCCTTaagCtTgCggAATG-3') (Mutated bases are shown as lowercase letters). Then using this 101 bp mutant fragment for a forward primer, we amplified a 1273 bp mutant fragment using a reverse primer (5'-GTGCAGGGTTATGCATTGTT-3'). The 1273 bp mutant fragment was digested with *Bpu*1102I and *Eco*T22I, and then the fragment was inserted into the *Bpu*1102I/*Eco*T22I-digested pGL3-hADRP-4K vector.

Nucleotide sequences of these plasmids were confirmed by ABI PRISM^® ^310 Genetic Analyzer (Applied Biosystems).

### Cell culture

HepG2 cells were cultured in DMEM (Nacalai tesque) containing 10% heat-inactivated charcoal/dextran treated foetal bovine serum (FBS) (HyClone), 100 IU/ml penicillin and 100 μg/ml streptomycin. The tightly tet-regulatable HepG2 cell clone (HY-Toff) which was transfected with the pTet-off vector, was isolated as previously described [22]. HY-Toff cells were co-transfected with pBI-EGFP-hPPAR and pBabepuro using TransIT^®^-LT1 Transfection Reagents (Mirus). The cells were cultured in a medium containing 600 μg/ml G418 (Nacalai tesque), 1 μg/ml puromycin (Sigma) and 1 μg/ml Dox (Clontech). G418- and puromycin-resistant clones were isolated. These clones were further screened for well-inducible clones by checking the expression of EGFP using fluorescence microscopy and the expression of PPAR proteins using immunoblot analysis in the absence of Dox.

### Quantitative Real-Time PCR

Total RNA was isolated using an RNA preparation kit (Isogen; Nippon Gene Corp.). First strand cDNA was synthesized from 5 μg of total RNA of each cell sample using the SuperScript™ First-Strand Synthesis System for RT-PCR (Invitrogen) with oligo(dT)_12–18 _primer. The cDNAs were then used as templates for individual PCR reactions using specific primer sets (Table 2), which were designed by the Primer3 program written by the Whitehead Institute [43]. PCR reactions were carried out using QuantiTect™ SYBR^® ^Green PCR Kit (Qiagen). The quantitative PCR analysis was performed using the DNA Engine Opticon™ System (Bio-Rad Laboratories). Amplification specificity was verified by visualizing PCR products on an ethidium bromide-stained 3% agarose gel. Glyceraldehyde-3-phosphate dehydrogenase (GAPDH) was used for normalizing each expression data.

**Table 2:** Primers used for Real-Time PCR Analysis. Sequences of forward (For.) and reverse (Rev.) primer for each target are shown. Sequences are 5' to 3'.

Gene	Sequence	Size (bp)
ADRP	For. primer : TGAGATGGCAGAGAACGGTGTG	184
	Rev. primer : GGCATTGGCAACAATCTGAGT	
ANGPTL4	For. primer : GGGAGAGGCAGAGTGGACTATTT	96
	Rev. primer : TTACTGTCCAGCCTCCATCTGA	
CLAMP/PDZK1	For. primer : CTAAACTCTGCAGGCTGGCTAAA	101
	Rev. primer : GCCCTTCTGTACCTCTTTGATGA	
HADHA	For. primer : CAAGGGCTTCCTAGGTCGTAAAT	155
	Rev. primer : GGAACTGGATGTCTTCGTCTGAT	
HMGCS2	For. primer : GGAACCCATATGGAGAATGTGT	168
	Rev. primer : ATCGCTGCCAGCTTGCTT	
GAPDH	For. primer : TGGGTGTGAACCATGAGAAG	76
	Rev. primer : GCTAAGCAGTTGGTGGTGC	
L-FABP	For. primer : TTGCCACCATGAGTTTCTCCG	82
	Rev. primer : GGCAGACCGATTGCCTTCA	
MCAD	For. primer : TTCCAGAGAACTGTGGAGGTCTT	100
	Rev. primer : TCAATAGCAGTCTGAACCCCTGT	
PEPCK	For. primer : TGCATGAAAGGTCGCACCA	192
	Rev. primer : CACAGAATGGAGGCATTTGACA	
PPARα	For. primer : CTATCATTTGCTGTGGAGATCG	121
	Rev. primer : AAGATATCGTCCGGGTGGTT	
PPARδ	For. primer : GTCACACAACGCTATCCGTTT	143
	Rev. primer : AGGCATTGTAGATGTGCTTGG	
PPARγ1	For. primer : CGTGGCCGCAGATTTGAA	166
	Rev. primer : CTTCCATTACGGAGAGATCCAC	
PPARγ2	For. primer : GGTGAAACTCTGGGAGATTCT	102
	Rev. primer : CTCTGTGTCAACCATGGTCA	

### Immunoblot Analysis

Nuclear extracts were obtained as previously described [44]. Each nuclear extract (50 μg) was resolved by 10% SDS-PAGE, and electroblotted to nitrocellulose membranes. Western blot analyses were carried out using anti-human PPARα [H0723], PPARδ [K7701] (Perseus Proteomics Inc.) or PPARγ [E-8] antibodies (Santa Cruz). The signals were visualized with the ECL detection system (Amersham Biosciences).

### Affymetrix Oligonucleotide Microarray Analysis

HepG2-tet-off-hPPAR cells were cultured in the presence (Dox) or absence of Dox for 5 days. In the case of the absence of Dox, the cells were treated with PPAR ligands (100 μM fenofibric acid for PPARα (Feno), 100 nM GW501516 for PPARδ (GW) or 10 μM ciglitizone for PPARγ (Cig)) or vehicle (DMSO) for 24 h. Total RNA samples were prepared from these cells using an RNA preparation kit (Isogen). Hybridization samples were prepared according to the Affymetrix protocol as previously described [7]. Briefly, 10 μg total RNA was used to generate first-strand cDNA. After second-strand cRNA synthesis, biotinylated and amplified RNAs were purified using RNeasy (Qiagen) and quantitated by a spectrophotometer. Biotinylated cRNA samples were then hybridized to Affymetrix Human Genome U133A arrays. These arrays contain probe sets for >22,000 transcripts and EST clones. After hybridization, microarrays were washed, scanned, and analysed with the GeneChip^® ^software Microarray Suite (MAS) Ver.5.0 (Affymetrix). The criteria for selecting genes that were induced by ligand-activated PPARs were as follows: (1) in the presence of ligand, the average difference of gene was ≥ 100 and the gene represented as "presence"; (2) the ratio of the expression level in the presence of ligand to the expression level in the presence of Dox was greater than two; and (3) the ratio of the expression level in the presence of ligand to the expression level in the absence of ligand was greater than 1.5. Raw data are available at NCBI GEO, web page accession number GSE2699.

### Luciferase Assay

HepG2 cells were transfected using Lipofectamine™ 2000 (Invitrogen) according to the manufacturer's instructions. HepG2 cells (3 × 10^4 ^cells/well) were seeded in 96-well plates 14–18 h before transfection. The cells were transfected with 50 ng of human ADRP reporter plasmid, 50 ng of phRL-TK (Promega) and either 5 ng of pcDNA3, pcDNA3-hPPARα, pcDNA3-hPPARδ, pcDNA3-hPPARγ1 or the pcDNA3-hPPARγ2 expression vector. Twenty-four hours following transfection, the cells were incubated with a medium containing dimethylsulfoxide (DMSO) (vehicle), 100 μM fenofibric acid, 100 nM GW501516 or 10 μM ciglitizone. Following a period of 24 h, both firefly and *Renilla *luciferase activities were quantified using a Dual-Luciferase^® ^Reporter Assay System (Promega) according to manufacturer's instructions.

### Electrophoretic Mobility Shift Assay (EMSA)

Human PPARα, human PPARδ, human PPARγ2 and human RXRα proteins were prepared using the IMPACT™-CN system (New England Biolabs). Human PPARγ1 protein was synthesized *in vitro *using the TNT^® ^Quick Coupled Transcription/Translation Systems (Promega). Double-strand oligonucleotides were labelled with [α-^32^P]dCTP and a Klenow fragment and were used as probes. PPARα, PPARδ, PPARγ1, PPARγ2 and/or RXRα proteins were incubated with ^32^P-labelled probe in a total volume of 12.5 μl binding buffer (10 mM Tris-HCl (pH 7.5), 5% glycerol, 1 mM DTT, 1 mM EDTA, 1 μg poly (dI-dC), 30 μg BSA) at room temperature for 30 min, followed by an incubation at 4°C for 30 min. Supershift assays were performed by adding antibodies 1 h before incubation with an oligonucleotide probe at room temperature. All monoclonal antibodies (PPARα [H0723], PPARδ [K9418], PPARγ [A3408A]) were obtained from Perseus Proteomics. In competition studies, the proteins were pre-incubated with 10- or 100-fold molar excess of unlabelled wild-type or mutant oligonucleotides. Protein-DNA complexes were resolved on a 5% nondenaturing polyacrylamide gel in 1 × TAE buffer. The loaded gel was fixed with 10% methanol and 10% acetic acid, and then the gel was dried and autoradiographed. Double-stranded oligonucleotides composed of the following sequences were used for the binding and competition assays: human ADRP PPRE wild type, 5'-GCATTTTGTAGGTGAAAGGGCGAGAGTC-3'; ADRP PPRE mutant, 5'-GCATTccGcAaGcttAAGGGCGAGAGTC-3', and rat acyl-CoA oxidase (ACO) PPRE wild type, 5'-GCGGACCAGGACAAAGGTCACGTTC-3' (Mutated bases are shown as lowercase letters).

### Chromatin Immunoprecipitation (ChIP) Assay

HepG2-tet-off-hPPAR cells were cultured in the absence of Dox for 5 days. The cells were treated with PPAR ligands (100 μM fenofibric acid for PPARα, 100 nM GW501516 for PPARδ, or 10 μM troglitazone for PPARγ) for 8 h. Cells were fixed *in vivo *at room temperature for 10 min by the addition of formaldehyde at a final concentration of 1% directly onto the cell culture media. Fixation was completed following the addition of glycine with a 0.125 M final concentration and the incubation was continued for a further 5 min. The cells were washed twice using ice-cold phosphate-buffered saline and collected. The cell pellets were washed with cell lysis buffer (10 mM Tris-HCl (pH 7.5), 10 mM NaCl, 3 mM MgCl_2_, 0.5% NP-40, and a protease inhibitor cocktail (Sigma)) three times, and dissolved in SDS lysis buffer (10 mM Tris-HCl (pH 7.5), 270 mM NaCl, 3 mM MgCl_2_, 1 mM CaCl_2_, 4% NP-40, 1.3% SDS, and a protease inhibitor cocktail) and remained on ice for 10 min. The cell lysates were sonicated to shear chromosomal DNA with an average length of 1000 bp. After centrifugation to remove insoluble materials, the chromatin solution was diluted 10-fold in an IP dilution buffer (20 mM Tris-HCl (pH 8.0), 150 mM NaCl, 2 mM EDTA, 1% Triton X-100, 0.01% SDS, and a protease inhibitor cocktail), and the diluted solution was pre-cleared with protein G Sepharose beads on a rotating wheel at 4°C for 1 h. Beads were removed by centrifugation and the supernatants were incubated with 2 μg of antibodies to PPARα (H-98, Santa Cruz), PPARδ (H-74, Santa Cruz), PPARγ (H-100, Santa Cruz), or RXRα (D-20, Santa Cruz) at 4°C overnight. For a negative control, pre-immune rabbit IgG (Santa Cruz) was incubated with the supernatant. The complexes were immunoprecipitated with protein G Sepharose beads. The beads were washed once with IP dilution buffer, twice with wash buffer 1 (20 mM Tris-HCl (pH 8.0), 150 mM NaCl, 2 mM EDTA, 1% Triton X-100, 0.1% SDS, and a protease inhibitor cocktail), once with wash buffer 2 (20 mM Tris-HCl (pH 8.0), 500 mM NaCl, 2 mM EDTA, 1% Triton X-100, 0.1% SDS, and a protease inhibitor cocktail), once with wash buffer 3 (10 mM Tris-HCl (pH 8.0), 1 mM EDTA, 0.25 M LiCl, 1% NP-40, 1% deoxycholate), and twice with TE buffer. Immune complexes were eluted from the beads in the elution buffer (25 mM Tris-HCl (pH 7.5), 5 mM EDTA, 0.5% SDS, 10 mM DTT) for 15 min. The proteins were removed from DNA by digesting with 1.5 mg/ml pronase at 42°C for 2 h. The crosslink was reversed by adding 5 M NaCl to a final concentration of 200 mM followed by incubation at 65°C for 6 h. The sample DNAs were then extracted with phenol-chloroform-isoamyl alcohol (25:24:1), precipitated with ethanol in the presence of glycogen, and resuspended in TE buffer. Similarly purified DNA fragments from the chromatin extracts (input) were used as a control for PCR reactions. Precipitated DNAs were analysed by PCR of 32 cycles using primers 5'-GCAAAAAGAAGCTTGCTCAG-3' and 5'-TGTTGCCATCTTCAGTGTTT-3' that flanked the PPRE of the ADRP promoter or primers 5'-ATGGTTGCCACTGGGGATCT-3' and 5'-TGCCAAAGCCTAGGGGAAGA-3' that are located about 6-kb upstream of the GAPDH promoter (negative control). PCR products were separated on a 2% agarose gel and stained with ethidium bromide.

## List of Abbreviations

ACO, acyl-CoA oxidase; ADRP, adipose differentiation-related protein; ANGPTL4, angiopoietin-like protein 4; CLAMP/PDZK1, C-terminal linking and modulating protein/PDZ domain containing 1; DMSO, dimethylsulfoxide; Dox, doxycycline; GAPDH, glyceraldehyde-3-phosphate dehydrogenase; HADHA, hydroxyacyl-Coenzyme A dehydrogenase/3-ketoacyl-Coenzyme A thiolase/enoyl-Coenzyme A hydratase (trifunctional protein), α subunit; HDL, high density lipoprotein; HMGCS2, 3-hydroxy-3-methylglutaryl-Coenzyme A synthase 2 (mitochondrial); L-FABP, fatty acid binding protein 1, liver; MCAD, acyl-Coenzyme A dehydrogenase, C-4 to C-12 straight chain; PEPCK, phosphoenolpyruvate carboxykinase 1; PPAR, peroxisome proliferator-activated receptor; PPRE, peroxisome proliferator responsive element; RXR, retinoid X receptor; Tet, tetracycline.

## Competing interests

The author(s) declare that they have no competing interests.

## Authors' contributions

K. Tachibana carried out all the experiments and prepared the manuscript. Y. Kobayashi performed the reporter gene assays. T. Tanaka participated in designing the experiments. Y. Kobayashi and A. Sugiyama generated the human ADRP promoter-reporter plasmids. Y. Kobayashi, M. Tagami, T. Katayama, C. Ueda, D. Yamasaki, K. Ishimoto, and M. Sumitomo assisted with cell culture and the establishment of the stable cell lines. Y. Kobayashi, M. Tagami, T. Katayama, C. Ueda, and Y. Uchiyama performed the immunoblot analyses. T. Kohro assisted with the analysis of the microarray data. J. Sakai, T. Hamakubo, T. Kodama, and T. Doi developed the idea for the study, and participated in its design and coordination. All authors read and approved the final manuscript.

## Supplementary Material

Additional File 1 **Changes in mRNA expression levels in HepG2-tet-off-hPPAR cell lines by ligands. **Microarray analyses were performed on HepG2-tet-off-hPPAR cells; the cells were cultured in the presence (Dox) or absence of Dox for 5 days. In the absence of Dox, the cells were treated with PPAR ligands (100 μM fenofibric acid for PPARα (Feno), 100 nM GW501516 for PPARδ (GW) or 10 μM ciglitizone for PPARγ (Cig)) or vehicle (DMSO) for 24 h. Gene expression profiles were compared between DMSO and Dox (DMSO *versus *Dox), ligands and Dox (Feno *versus *Dox, GW *versus *Dox, and Cig *versus *Dox were indicated in the case of PPARα, PPARδ and PPARγ, respectively) or ligands and DMSO (Feno *versus *DMSO, GW *versus *DMSO, and Cig *versus *DMSO were indicated in the case of PPARα, PPARδ and PPARγ, respectively). Average differences expressed the intensities of the mRNA levels in HepG2-tet-off-hPPARs cell lines. Samples were analysed using GeneChip^® ^software Microarray Suite (MAS) Ver.5.0 (Affymetrix). Click here for file

## References

[B1] Mangelsdorf DJ, Thummel C, Beato M, Herrlich P, Schutz G, Umesono K, Blumberg B, Kastner P, Mark M, Chambon P, Evans RM (1995). The nuclear receptor superfamily: the second decade. Cell.

[B2] Desvergne B, Wahli W (1999). Peroxisome proliferator-activated receptors: nuclear control of metabolism. Endocr Rev.

[B3] Willson TM, Brown PJ, Sternbach DD, Henke BR (2000). The PPARs: from orphan receptors to drug discovery. J Med Chem.

[B4] Mandard S, Muller M, Kersten S (2004). Peroxisome proliferator-activated receptor alpha target genes. Cell Mol Life Sci.

[B5] Corton JC, Lapinskas PJ, Gonzalez FJ (2000). Central role of PPARalpha in the mechanism of action of hepatocarcinogenic peroxisome proliferators. Mutat Res.

[B6] Cheung C, Akiyama TE, Ward JM, Nicol CJ, Feigenbaum L, Vinson C, Gonzalez FJ (2004). Diminished hepatocellular proliferation in mice humanized for the nuclear receptor peroxisome proliferator-activated receptor alpha. Cancer Res.

[B7] Tanaka T, Yamamoto J, Iwasaki S, Asaba H, Hamura H, Ikeda Y, Watanabe M, Magoori K, Ioka RX, Tachibana K, Watanabe Y, Uchiyama Y, Sumi K, Iguchi H, Ito S, Doi T, Hamakubo T, Naito M, Auwerx J, Yanagisawa M, Kodama T, Sakai J (2003). Activation of peroxisome proliferator-activated receptor delta induces fatty acid beta-oxidation in skeletal muscle and attenuates metabolic syndrome. Proc Natl Acad Sci U S A.

[B8] Dressel U, Allen TL, Pippal JB, Rohde PR, Lau P, Muscat GE (2003). The peroxisome proliferator-activated receptor beta/delta agonist, GW501516, regulates the expression of genes involved in lipid catabolism and energy uncoupling in skeletal muscle cells. Mol Endocrinol.

[B9] Wang YX, Lee CH, Tiep S, Yu RT, Ham J, Kang H, Evans RM (2003). Peroxisome-proliferator-activated receptor delta activates fat metabolism to prevent obesity. Cell.

[B10] Tan NS, Michalik L, Noy N, Yasmin R, Pacot C, Heim M, Fluhmann B, Desvergne B, Wahli W (2001). Critical roles of PPAR beta/delta in keratinocyte response to inflammation. Genes Dev.

[B11] Chawla A, Lee CH, Barak Y, He W, Rosenfeld J, Liao D, Han J, Kang H, Evans RM (2003). PPARdelta is a very low-density lipoprotein sensor in macrophages. Proc Natl Acad Sci U S A.

[B12] Tontonoz P, Hu E, Graves RA, Budavari AI, Spiegelman BM (1994). mPPAR gamma 2: tissue-specific regulator of an adipocyte enhancer. Genes Dev.

[B13] Tontonoz P, Hu E, Spiegelman BM (1994). Stimulation of adipogenesis in fibroblasts by PPAR gamma 2, a lipid-activated transcription factor. Cell.

[B14] Vidal-Puig A, Jimenez-Linan M, Lowell BB, Hamann A, Hu E, Spiegelman B, Flier JS, Moller DE (1996). Regulation of PPAR gamma gene expression by nutrition and obesity in rodents. J Clin Invest.

[B15] Chao L, Marcus-Samuels B, Mason MM, Moitra J, Vinson C, Arioglu E, Gavrilova O, Reitman ML (2000). Adipose tissue is required for the antidiabetic, but not for the hypolipidemic, effect of thiazolidinediones. J Clin Invest.

[B16] Memon RA, Tecott LH, Nonogaki K, Beigneux A, Moser AH, Grunfeld C, Feingold KR (2000). Up-regulation of peroxisome proliferator-activated receptors (PPAR-alpha) and PPAR-gamma messenger ribonucleic acid expression in the liver in murine obesity: troglitazone induces expression of PPAR-gamma-responsive adipose tissue-specific genes in the liver of obese diabetic mice. Endocrinology.

[B17] Bedoucha M, Atzpodien E, Boelsterli UA (2001). Diabetic KKAy mice exhibit increased hepatic PPARgamma1 gene expression and develop hepatic steatosis upon chronic treatment with antidiabetic thiazolidinediones. J Hepatol.

[B18] Kliewer SA, Umesono K, Noonan DJ, Heyman RA, Evans RM (1992). Convergence of 9-cis retinoic acid and peroxisome proliferator signalling pathways through heterodimer formation of their receptors. Nature.

[B19] Hsu MH, Palmer CN, Song W, Griffin KJ, Johnson EF (1998). A carboxyl-terminal extension of the zinc finger domain contributes to the specificity and polarity of peroxisome proliferator-activated receptor DNA binding. J Biol Chem.

[B20] Gervois P, Chopin-Delannoy S, Fadel A, Dubois G, Kosykh V, Fruchart JC, Najib J, Laudet V, Staels B (1999). Fibrates increase human REV-ERBalpha expression in liver via a novel peroxisome proliferator-activated receptor response element. Mol Endocrinol.

[B21] Schoonjans K, Staels B, Auwerx J (1996). Role of the peroxisome proliferator-activated receptor (PPAR) in mediating the effects of fibrates and fatty acids on gene expression. J Lipid Res.

[B22] Huang Y, Uchiyama Y, Fujimura T, Kanamori H, Doi T, Takamizawa A, Hamakubo T, Kodama T (2001). A human hepatoma cell line expressing hepatitis c virus nonstructural proteins tightly regulated by tetracycline. Biochem Biophys Res Commun.

[B23] Gavrilova O, Haluzik M, Matsusue K, Cutson JJ, Johnson L, Dietz KR, Nicol CJ, Vinson C, Gonzalez FJ, Reitman ML (2003). Liver peroxisome proliferator-activated receptor gamma contributes to hepatic steatosis, triglyceride clearance, and regulation of body fat mass. J Biol Chem.

[B24] Kersten S, Seydoux J, Peters JM, Gonzalez FJ, Desvergne B, Wahli W (1999). Peroxisome proliferator-activated receptor alpha mediates the adaptive response to fasting. J Clin Invest.

[B25] Leone TC, Weinheimer CJ, Kelly DP (1999). A critical role for the peroxisome proliferator-activated receptor alpha (PPARalpha) in the cellular fasting response: the PPARalpha-null mouse as a model of fatty acid oxidation disorders. Proc Natl Acad Sci U S A.

[B26] Patsouris D, Mandard S, Voshol PJ, Escher P, Tan NS, Havekes LM, Koenig W, Marz W, Tafuri S, Wahli W, Muller M, Kersten S (2004). PPARalpha governs glycerol metabolism. J Clin Invest.

[B27] Way JM, Harrington WW, Brown KK, Gottschalk WK, Sundseth SS, Mansfield TA, Ramachandran RK, Willson TM, Kliewer SA (2001). Comprehensive messenger ribonucleic acid profiling reveals that peroxisome proliferator-activated receptor gamma activation has coordinate effects on gene expression in multiple insulin-sensitive tissues. Endocrinology.

[B28] Bernal-Mizrachi C, Weng S, Feng C, Finck BN, Knutsen RH, Leone TC, Coleman T, Mecham RP, Kelly DP, Semenkovich CF (2003). Dexamethasone induction of hypertension and diabetes is PPAR-alpha dependent in LDL receptor-null mice. Nat Med.

[B29] Kocher O, Comella N, Tognazzi K, Brown LF (1998). Identification and partial characterization of PDZK1: a novel protein containing PDZ interaction domains. Lab Invest.

[B30] Ikemoto M, Arai H, Feng D, Tanaka K, Aoki J, Dohmae N, Takio K, Adachi H, Tsujimoto M, Inoue K (2000). Identification of a PDZ-domain-containing protein that interacts with the scavenger receptor class B type I. Proc Natl Acad Sci U S A.

[B31] Silver DL (2002). A carboxyl-terminal PDZ-interacting domain of scavenger receptor B, type I is essential for cell surface expression in liver. J Biol Chem.

[B32] Acton S, Rigotti A, Landschulz KT, Xu S, Hobbs HH, Krieger M (1996). Identification of scavenger receptor SR-BI as a high density lipoprotein receptor. Science.

[B33] Murao K, Terpstra V, Green SR, Kondratenko N, Steinberg D, Quehenberger O (1997). Characterization of CLA-1, a human homologue of rodent scavenger receptor BI, as a receptor for high density lipoprotein and apoptotic thymocytes. J Biol Chem.

[B34] Margeli A, Kouraklis G, Theocharis S (2003). Peroxisome proliferator activated receptor-gamma (PPAR-gamma) ligands and angiogenesis. Angiogenesis.

[B35] Shi Y, Hon M, Evans RM (2002). The peroxisome proliferator-activated receptor delta, an integrator of transcriptional repression and nuclear receptor signaling. Proc Natl Acad Sci U S A.

[B36] Krogsdam AM, Nielsen CA, Neve S, Holst D, Helledie T, Thomsen B, Bendixen C, Mandrup S, Kristiansen K (2002). Nuclear receptor corepressor-dependent repression of peroxisome-proliferator-activated receptor delta-mediated transactivation. Biochem J.

[B37] Brasaemle DL, Barber T, Wolins NE, Serrero G, Blanchette-Mackie EJ, Londos C (1997). Adipose differentiation-related protein is an ubiquitously expressed lipid storage droplet-associated protein. J Lipid Res.

[B38] Targett-Adams P, McElwee MJ, Ehrenborg E, Gustafsson MC, Palmer CN, McLauchlan J (2005). A PPAR response element regulates transcription of the gene for human adipose differentiation-related protein. Biochim Biophys Acta.

[B39] Cheng L, Ding G, Qin Q, Huang Y, Lewis W, He N, Evans RM, Schneider MD, Brako FA, Xiao Y, Chen YE, Yang Q (2004). Cardiomyocyte-restricted peroxisome proliferator-activated receptor-delta deletion perturbs myocardial fatty acid oxidation and leads to cardiomyopathy. Nat Med.

[B40] Oliver WRJ, Shenk JL, Snaith MR, Russell CS, Plunket KD, Bodkin NL, Lewis MC, Winegar DA, Sznaidman ML, Lambert MH, Xu HE, Sternbach DD, Kliewer SA, Hansen BC, Willson TM (2001). A selective peroxisome proliferator-activated receptor delta agonist promotes reverse cholesterol transport. Proc Natl Acad Sci U S A.

[B41] Giddings SJ, Clarke SE, Gibson GG (1997). CYP4A1 gene transfection studies and the peroxisome proliferator-activated receptor: development of a high-throughput assay to detect peroxisome proliferators. Eur J Drug Metab Pharmacokinet.

[B42] Tanaka T, Takeno T, Watanabe Y, Uchiyama Y, Murakami T, Yamashita H, Suzuki A, Aoi R, Iwanari H, Jiang SY, Naito M, Tachibana K, Doi T, Shulman AI, Mangelsdorf DJ, Reiter R, Auwerx J, Hamakubo T, Kodama T (2002). The generation of monoclonal antibodies against human peroxisome proliferator-activated receptors (PPARs). J Atheroscler Thromb.

[B43] Rozen S, Skaletsky H (2000). Primer3 on the WWW for general users and for biologist programmers. Methods Mol Biol.

[B44] Minami T, Tachibana K, Imanishi T, Doi T (1998). Both Ets-1 and GATA-1 are essential for positive regulation of platelet factor 4 gene expression. Eur J Biochem.

